# *Chlamydia trachomatis*-specific interferon-γ-producing CD8 T-cells are associated with lower chlamydia bacterial load in reinfected women

**DOI:** 10.1093/immhor/vlaf004

**Published:** 2025-04-01

**Authors:** William M Geisler, Shara B Legg, David C Moylan, Kanupriya Gupta, Barbara Van Der Pol, Hemant Tiwari, Steffanie Sabbaj

**Affiliations:** Department of Medicine, University of Alabama at Birmingham, Birmingham, AL, United States; Department of Medicine, University of Alabama at Birmingham, Birmingham, AL, United States; Department of Medicine, University of Alabama at Birmingham, Birmingham, AL, United States; Department of Medicine, University of Alabama at Birmingham, Birmingham, AL, United States; Department of Medicine, University of Alabama at Birmingham, Birmingham, AL, United States; Department of Biostatistics, University of Alabama at Birmingham, Birmingham, AL, United States; Department of Medicine, University of Alabama at Birmingham, Birmingham, AL, United States

**Keywords:** bacterial infections, cellular immune response, T cells

## Abstract

This study aimed to better understand the importance of CD8 T cell responses in protective immunity to chlamydia. In women evaluated for reinfection at a 3-month follow-up visit after treatment for chlamydia, the presence or magnitude of *Chlamydia trachomatis*-specific CD8 interferon-gamma (IFN-γ) responses to Momp and Pgp3 peptide pools was not associated with reinfection status, despite having an increased frequency of responses compared to *C. trachomatis* CD4-specific T cells. However, reinfected women with detectable interferon-gamma (IFN-γ)-producing CD8 T cells had lower *C. trachomatis* bacterial load compared to women without these CD8 T cell responses. Moreover, the frequency of IFN-γ-producing CD8 T cells was inversely associated with *C. trachomatis* bacterial load. We further determined that *C. trachomatis*-specific IFN-γ-producing CD8 T cells were predominately late differentiated effector memory T cells that re-expressed CD45RA (Temra; CCR7-CD45RA+) or effector memory T cells (Tem; CCR7-CD45RA-). Together, these data support the concept that CD8 T cells may contribute to protective immunity against chlamydia in women.

## Introduction

*Chlamydia trachomatis* infection is still highly prevalent^1^ despite decades of chlamydia control efforts. This suggests that a biomedical intervention, such as a chlamydial vaccine, is needed to reduce the burden of disease since chlamydia contributes to reproductive sequelae.^2^ Knowledge related to adaptive immunity to chlamydia in humans is essential for advancing research progress on vaccines for chlamydia prevention. Murine and human chlamydia studies have often generated conflicting results. Studies show *Chlamydia*-specific interferon-gamma (IFN-γ)-producing CD4 T cells are essential in protective immunity;^3–5^ however, the role of CD8 T cells in immunity to chlamydia is unclear. Murine studies show CD8 T cells are not essential,^3^ but a macaque trachoma vaccine study showed CD8 T cells were necessary for protection.^6^ Our prior study on adaptive T cell responses in peripheral blood mononuclear cells (PBMCs) from women treated for chlamydia found a significantly higher frequency of *C. trachomatis*-specific CD4, but not CD8, IFN-γ responses in women without reinfection^5^; however, *C. trachomatis* antigens used were optimum for inducing CD4 T cell responses, likely skewing responses to CD4 T cells and limiting evaluation of CD8 T cell responses.

In the current study, we reassessed CD8 T cell responses in protective immunity to chlamydia in women from the same cohort previously evaluated for reinfection^5^ by using *C. trachomatis* plasmid gene protein 3 (Pgp3) and major outer membrane protein (MOMP) peptide pools including peptide lengths shown to represent a good compromise for stimulating both CD4 and CD8 T cells.^7^ Given that we previously showed reinfected women had lower *C. trachomatis* loads at the time of reinfection,^8^ we also expanded our earlier work by investigating association of *C. trachomatis*-specific T cell responses with bacterial load, which may serve as a marker of vaccine efficacy in clinical studies. Finally, we identified the T cell memory subsets contributing to *C. trachomatis*-specific IFN-γ.

## Results

### Baseline participant characteristics

For the 76 women studied, median age was 22 (range 16 to 29), all reported Black race, and 75 (99%) reported non-Hispanic ethnicity. Prior chlamydia was reported or documented for 37 (49%), and 32 (43%) used hormonal contraception. Vaginal infections were highly prevalent: bacterial vaginosis was diagnosed in 30%, candidiasis in 14%, and trichomoniasis in 7%.

### *C. trachomatis*-specific CD8 responses and relation to reinfection

**Figure 1.**
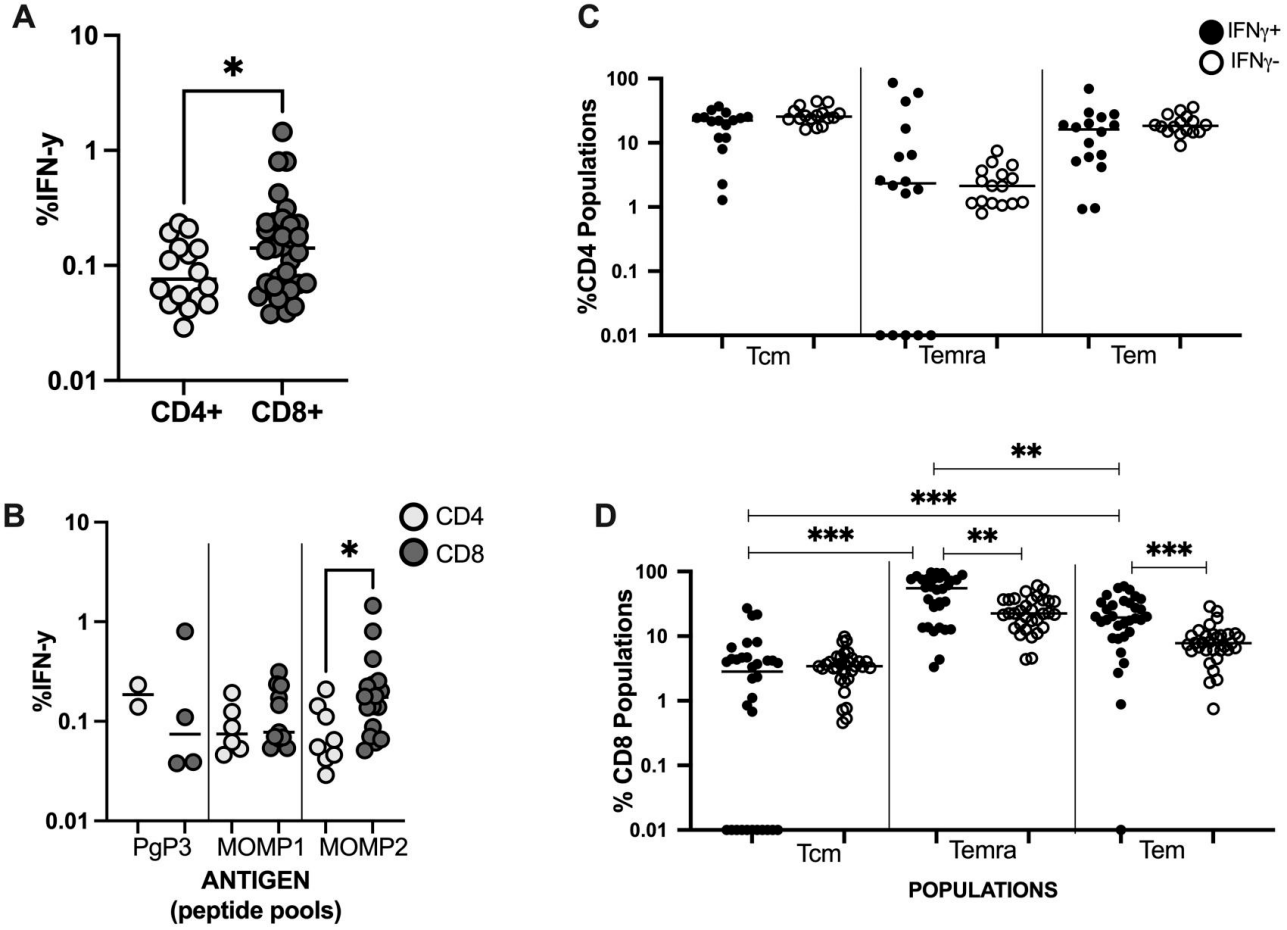
Distribution of *Chlamydia trachomatis*(CT)-specific IFN-γ-producing CD4 and CD8 T cells and associated memory cell populations in peripheral blood mononuclear cells from women seen for a 3-month follow-up visit after treatment for chlamydia (n = 76). Comparison of the frequency of all CD4 and CD8 T cells producing IFN-γ in response to: (A) all CT antigens tested and (B). to different CT antigens tested. Comparison of the frequency of CT-specific memory T cell subsets producing IFN-γ among: (C) CD4 T cells and (D) CD8 T cells. Memory T cell subsets: central memory T cells (Tcm; CCR7+CD45RA-); late differentiated effector memory T cells that re-express CD45RA (Temra; CCR7-CD45RA+); and effector memory T cells (Tem; CCR7-CD45RA-). Median frequencies are represented by a horizontal line. (A) There was a higher frequency of CD8 T cells producing IFN-γ in response to all CT antigens tested compared to CD4 T cells (*P = 0.0498). (B) There was a higher frequency of CD8 T cells producing IFN-γ in response to CT MOMP2 peptides compared to CD4 T cells (*P = 0.0133). (D) There was a higher frequency of both CD8 Temra and CD8 Tem that produced IFN-γ vs. did not (**P =* *.002 and ***P = 0.0001, respectively), and the frequency of IFN-γ-producing CD8 T cells was significantly higher for both Tem and Temra vs. Tcm (***P <* *0.0001 for both); among effector populations, frequency was significantly higher for Temra vs. Tem (**P =* *0.0027). Statistical significance was analyzed using the Mann-Whitney *U* test and Wilcoxon signed-rank matched-pairs test as appropriate.

We aimed to investigate CD8 T cell immunity in PBMCs from women seen at a 3 month follow-up visit after chlamydia treatment who did vs. did not have chlamydia reinfection at follow-up. We specifically designed this study to detect both CD4 and CD8 T cell responses by using overlapping peptide pools (see methods). Our data demonstrated that *C. trachomatis*-specific CD8 IFN-γ tumor necrosis factor-alpha (TNF-α and dual IFN-γ/Granzyme B responses to any of the *C. trachomatis* peptide pools were detected (positive, see methods) in PBMCs from 38%, 21%, and 33% of women, respectively (data not shown). Consistent with our previous findings,^5^ a *C. trachomatis*-specific CD8 IFN-γ or TNF-α response was not associated with reinfection status (*P* > 0.1); a CD8 dual IFN-γ/Granzyme B response was also not associated with reinfection status (*P* > 00.1) (data not shown). Among women with a *C. trachomatis*-specific CD8 IFN-γ, TNF-α, or dual IFN-γ/Granzyme B response, magnitude of response was also not associated with reinfection status (*P* > 00.1) (data not shown). *C. trachomatis*-specific CD8 T cells were more abundant than *C. trachomatis*-specific CD4 T cells and there was a higher frequency of CD8 T cells producing IFN-γ in response to all *C. trachomatis* antigens tested compared to CD4 T cells (median 0.142% vs. 0.076%, *P =* 0.0498; Fig. 1A), but there were no significant differences in frequency of *C. trachomatis*-specific CD4 vs. CD8 T cells producing TNF-α *P* > 00.1) or dual IFN-γ/Granzyme B (*P =* 0.0821; higher for CD8) (data not shown). Analyses of IFN-γ-producing cell frequency by specific antigen revealed a significantly higher frequency of CD8 T cells producing IFN-γ in response to MOMP2 peptides compared to CD4 T cells (median 0.174% vs. 0.06%, *P* = 0.0145) (Fig. 1B).

### Association between *C. trachomatis*-specific CD4 and CD8 IFN-γ and bacterial load

**Figure 2.**
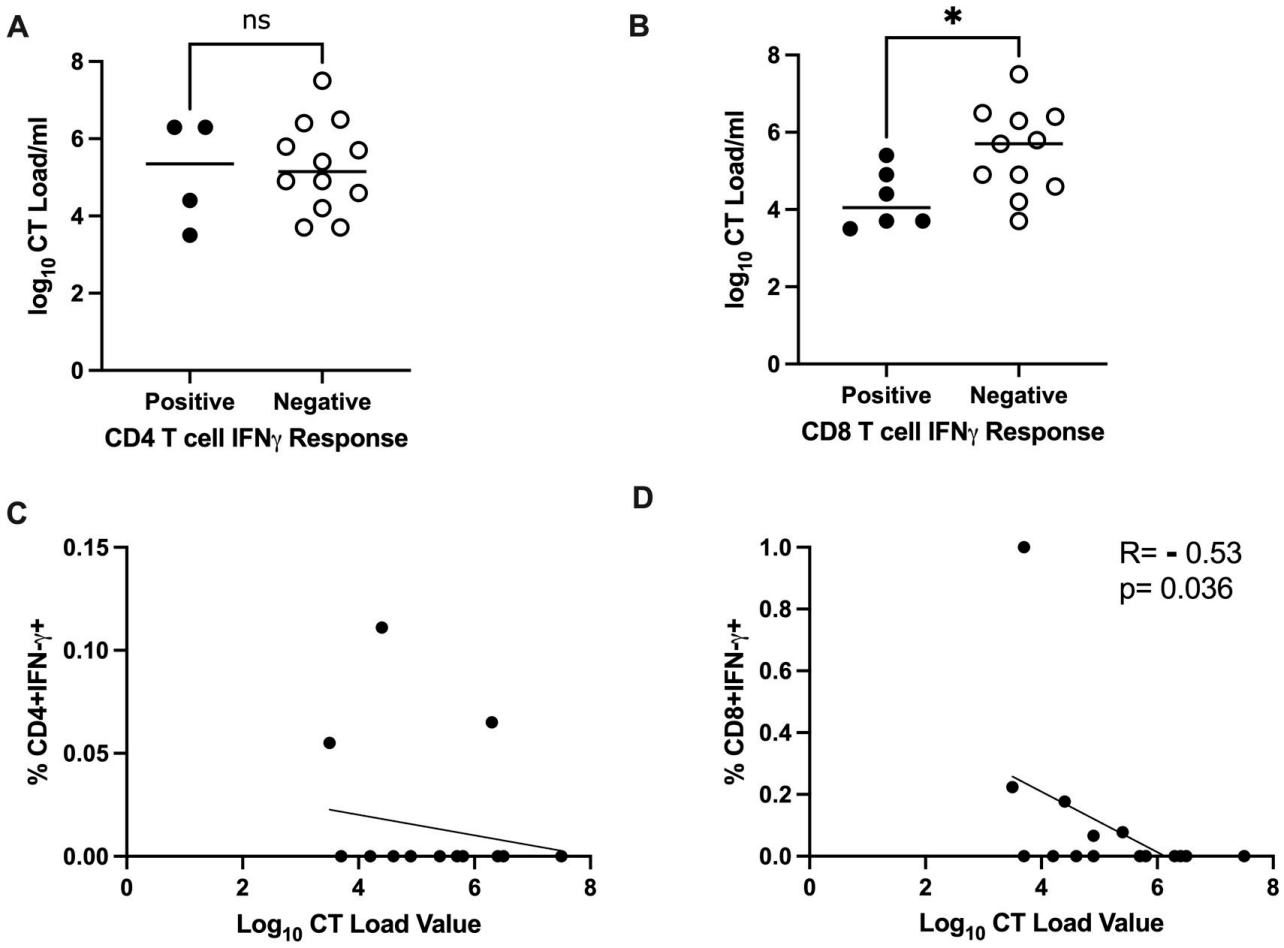
Association between *Chlamydia trachomatis*-specific IFN-γ-producing peripheral blood CD4 and CD8 T cells and *C. trachomatis* (CT) bacterial load among women with chlamydia reinfection at a 3-month follow-up visit after treatment for chlamydia (n = 17). CT bacterial load (log_10_ bacteria/ml) in women with a positive vs. negative CT-specific IFN-γ response in (A) CD4 T cells and (B) CD8 T cells. Median frequencies are represented by a horizontal line. Correlation of CT bacterial load (log_10_ bacteria/ml) with frequency of CT-specific IFN-γ-producing (C) CD4 T cells and (D) CD8 T cells. (*B*) Median bacterial load was lower in women with a CT*-*specific CD8 IFN-γ response vs. in those without a response (*P = 0.0338). (D) An inverse correlation was observed for frequency of CD8 T cells producing IFN-γ and bacterial load (r_s_ = −0.5312; *P *=* *0.0362). Statistical significance was analyzed using the Mann-Whitney *U* test or Spearman's rank correlation test as appropriate.

As a *C. trachomatis-*specific CD8 IFN-γ response was not associated with reinfection status, but these CD8 T cells predominated (compared to their CD4 counterparts), we sought to assess if CD8 IFN-γ may still contribute to protective immunity by assessing its association with bacterial load in women who had reinfection at their follow-up visit; for this analysis, we grouped the women by whether or not they had at least one positive response to the *C. trachomatis* antigens as detected by the production of IFNγ by CD4 (Fig. 2A) or CD8 T cells (Fig. 2B) vs. log_10_  *C. trachomatis* load. Our results showed that the median bacterial load (log_10_ bacteria/ml) was significantly lower in women with a *C. trachomatis-*specific CD8 IFN-γ response to any antigen versus in those without a response (log_10_ 4.05 vs. 5.3, *P* = 0.0338), but did not significantly differ based on CD4 IFN-γ response (*P* > 00.1) (Fig. 2A and B). Bacterial load also did not significantly differ based on CD4 or CD8 TNF-α or dual IFN-γ/Granzyme B responses (data not shown). We further analyzed the data by taking the total frequency of all antigens that met criteria for a positive response for each individual for the CD4 (Fig. 2C) or CD8 (Fig. 2D) IFNγ producing T cells and plotted this against the log_10_  *C. trachomatis* load/ml of serum. These results demonstrated that a statistically significant inverse correlation was observed only for the frequency of CD8 T cells producing IFN-γ versus bacterial load (*P =* 0.0342) that was not observed for IFN-γ-producing CD4 T cells (*P* > 0.1) (Fig. 2C and D).

### *C. trachomatis*-specific IFN-γ production among CD4 and CD8 memory subsets

Given we previously showed that *C. trachomatis*-specific CD4 IFN-γ was detected more often in women without reinfection^5^ and in the current study that *C. trachomatis*-specific CD8 IFN-γ was associated with lower bacteria load, we next wanted to determine if there were differences in the memory CD4 and CD8 T cell subsets in relation to *C. trachomatis*-specific IFN-γ-production. This analysis was done by gating on CD4 or CD8 T cells producing or not producing IFN-γ after stimulation with one of the *C. trachomatis* peptide pools tested. Followed by this gate, memory cell gates where placed to determine the following cell populations utilizing the lymph node homing receptor, CCR7 and the memory marker CD45RA (Fig. S1): central memory (Tcm; CCR7+CD45RA-), late differentiated effector memory T cells that re-expressed CD45RA (Temra; CCR7-CD45RA+), and effector memory (Tem; CCR7-CD45RA-). For CD4 T cells, there was no significant difference in frequency of Tcm, Temra, or Tem that did vs. did not produce IFN-γ in response to *C. trachomatis* antigens (*P =* 0.0675, *P* > 00.1, and *P* > 00.1, respectively; Fig. 1C). Conversely, for CD8 T cells, there was a higher frequency of Temra and Tem that produced IFN-γ vs. did not (*P =* 0.002 and *P =* 0.0001, respectively) but not Tcm (*P* > 0.1) (Fig. 1D). We also noted that the frequency of IFN-γ-producing CD8 T cells was significantly higher for both Tem and Temra vs. Tcm (both *P* < 0.0001) and among effector populations, the frequency was significantly higher for Temra vs. Tem (*P =* 0.0027) (Fig. 1D).

## Discussion

Murine and human studies show *Chlamydia*-specific CD8 T cell responses are induced following genital infection,^3^^,^^5^ but their importance in protective immunity is not clear. While murine chlamydia studies conclude CD8 T cell responses are not essential for immunity, some CD8 T cell clones and lines appear to provide modest immune protection in naïve mice, primarily through IFN-γ rather than cytotoxicity.^3^ Further, a macaque trachoma vaccine study showed that depletion of CD8 T cells in protected macaques completely abrogated protective immunity,^6^ and a study on intranasal immunization in mice showed that immune protection of genital mucosa was mediated by both CD4 and CD8 T cells producing IFN-γ.^9^ These studies support the notion that CD8 T cell responses contribute in protective immunity to chlamydia.

We aimed to investigate CD8 T cell immunity to chlamydia in women with recent chlamydial infection by utilizing overlapping peptide pools from *C. trachomatis* Pgp-3 and also MOMP peptides recognized by CD8 T cells,^10^^,^^11^ ensuring adequate detection of *C. trachomatis*-specific CD8 T cell responses and addressing a limitation of our earlier study of CD8 responses.^5^ We recapitulated our prior observation that *C. trachomatis*-specific CD8 T cell IFN-γ was not associated with absence of reinfection,^5^ and we found that *C. trachomatis*-specific CD8 T cell responses where more abundant than CD4 T cell responses.^12^ In addition, we demonstrated that *C. trachomatis*-specific CD8 T cell IFN-γ was associated with lower *C. trachomatis* bacterial load among reinfected women. These data, together with our previous study demonstrating that increased numbers of CD8 epitopes relative to CD4 epitopes are associated with effective immune responses against *C. trachomatis,*^12^ support the concept that CD8 T cell IFN-γ may contribute to protective immunity in humans. This idea is also supported by a study in macaques that demonstrated that animals vaccinated with a regimen using DNA priming and protein boosting with CTH522/CAF01 not only generated a *C. trachomatis*-specific CD8 T cell response (in addition to a CD4 response and IgG) but showed accelerated clearance of *C. trachomatis* infection.^13^

Given that both CD4 and CD8 T cells producing IFN-γ appear to be associated with protective immunity surrogates, we sought to determine the memory T cell subsets that contributed the *C. trachomatis*-specific IFN-γ. We found that *C. trachomatis*-specific IFN-γ producing CD8 T cells predominated relative to IFN-γ producing CD4 T cells when we used antigens that were able to bind both class I and class II HLA and thus stimulate responses from both CD4 and CD8 T cells. We noted that these IFN-γ producing CD8 T cells were mostly effector T cells, with a significantly greater proportion of Temra vs. Tem. Ibana et al. evaluated CD8 T cell subpopulations in PBMCs of a smaller cohort of women who were either *C. trachomatis* positive or negative and also found effector memory subpopulations predominated, yet a greater proportion were Tem vs. Temra.^14^ A key distinction between the studies that could account for difference in distribution of Tem vs. Temra was that our study delineated *C. trachomatis*-specific IFN-γ-producing memory subpopulations vs. those not producing *C. trachomatis*-specific IFN-γ and all women in our study had chlamydial infection about 3 months prior (when enrolled), and thus IFN-γ responses we measured likely reflected an adaptive *C. trachomatis*-specific response. Given that effector memory T cells can home to peripheral tissue sites of infection and aid in pathogen clearance, our finding that CD8 effector memory subsets dominated the *C. trachomatis*-specific IFN-γ responses when compared to CD4 T cells in which Tcm predominated, further supports the capacity of CD8 T cells to impact chlamydia clearance.

Our study was limited in not having mucosal mononuclear cells to compare *C. trachomatis*-specific IFN-γ-producing memory subpopulations in mucosal cells vs. those in PBMCs, which the Ibana et al. study showed a much higher proportion of CD8 Tem in endocervical mononuclear cells vs. PBMCs.^14^ Furthermore, our findings reflect ex vivo stimulation of PBMCs with *C. trachomatis* antigens, and it is possible that vaccine challenge with *C. trachomatis* antigens and an adjuvant may elicit in vivo CD4 and CD8 T cell IFN-γ responses of higher frequency and magnitude that have greater impact on immune protection, and may have differences in proportions of memory cell populations compared to our study. Finally, our study is hampered by the limited sample size.

In summary, we found that presence or magnitude of a CD8 IFN-γ response was not associated with chlamydia reinfection status, but CD8 IFN-γ was associated with lower bacterial load among reinfected women and that most *C. trachomatis*-specific IFN-γ-producing CD8 T cells constituted effector memory cells, mostly Temra. Collectively these studies suggest that CD8 T cells likely play a role in protective immunity to chlamydia, but temporal differences between CD8 and CD4 T cells in this protection are likely.

## Methods

### Sex as a biological variable

The current study used stored specimens from a cohort study that investigated immune responses in women with chlamydia. Women were the focus of the cohort study because chlamydia is more prevalent in women than men, and women are disproportionately impacted by chlamydia-associated morbidity, including reproductive and perinatal sequelae. It is unknown whether the findings are relevant for men.

### Study population

We tested stored PBMCs from women in the same study cohort that we had previously evaluated for T cell responses.^5^ Briefly, nonpregnant women seen at a health department sexual health clinic for management of a positive screening *C. trachomatis* nucleic acid amplification test (NAAT) were enrolled, received directly-observed azithromycin, and returned for a 3-month follow-up visit where reinfection status was determined using *C. trachomatis* NAAT.^5^ Of 93 women tested at follow-up,^5^ stored PBMCs were available for 74. We also included 3-month follow-up visit PBMCs for 2 additional women with reinfection that were not available in the earlier study bringing the current study total to 76 women: 26 with reinfection and 50 without reinfection.

### *C. trachomatis* antigens

The following peptides were synthesized (NEP, Gardner, Massachusetts, USA) and used at 10 uM to stimulate T cells: (1) overlapping *C. trachomatis* Pgp3 peptides (15-mer peptides overlapping by 11 amino acids); (2) *C. trachomatis* MOMP peptides with coverage of published CD4 epitopes (17 to 20-mers) and CD8 epitopes (9-mers),^10^^,^^11^ referred to as MOMP 1; and (3) 15mer-peptides from MOMP that did not overlap with any other human *Chlamydia* species’ MOMP, referred to as MOMP 2. Staphylococcal enterotoxin B (Sigma-Aldrich, St Louis, Missouri, USA) at 1 ug/ml was used as positive control and media spiked with 1% dimethyl sulfoxide as negative control.

### Intracellular cytokine staining

Intracellular cytokine staining (ICS) was performed as previously described.^15^ Briefly, anti-CD28 and anti-CD49d (each 1ug/ml; BD Biosciences, San Jose, California, USA), 50 U/ml of Benzonase (Novagen, Madison, Wisconsin, USA), 10 ug/ml Monensin (GolgiStop, BD Biosciences) and antigen were added to PBMCs incubated at 37°C and 5% CO_2_ for 6 hours, and placed at 4°C overnight. The following day, fluorescent LIVE/DEAD fixable Dead Cell Stain (Molecular probes, Invitrogen, California, USA) and cell-surface markers were added (Table S1). Cells were permeabilized with Cytofix/cytoperm (BD Biosciences) for 20 minutes followed by ICS. At least 100,000 lymphocytes were acquired using a FACSymphony flow cytometer (BD Biosciences). Data were analyzed using FlowJo 10.8.1 (TreeStar, San Carlos, California, USA). Lymphocytes were analyzed by gating on CD45 followed by exclusion of dead cells (Fig. S1). We used Fisher’s exact test to compare the number of cytokine-producing cells between antigen-stimulated and unstimulated (media/1% DMSO) samples to determine if a response was positive (*P* < 0.05 and frequency ≥2-fold above negative control).

### Bacterial load quantification

Quantification was performed using the cobas *C. trachomatis*/*Neisseria gonorrhoeae* NAAT (Roche Diagnostics, Indianapolis, Indiana, USA) as previously described.^8^ Briefly, this NAAT has amplification targets on the *C. trachomatis* cryptic plasmid and genome, and bacterial load is estimated with a *C. trachomatis* calibrator that utilizes *C. trachomatis* reference strains with known bacterial loads. Of 26 women with reinfection in the current study, bacterial load data were available for 17.

### Statistical analyses

Analyses were performed using Stata (version 14.0; StataCorp, College Station, Texas, USA). Immune responses in paired vs. unpaired samples were analyzed using the Wilcoxon signed-rank matched-pairs test vs. Mann-Whitney *U* test, respectively. Correlation of IFN-γ response with bacterial load was analyzed using Spearman's rank correlation test. Differences with *P* < 0.05 were considered significant.

### Study approval

The study was approved by the University of Alabama at Birmingham Institutional Review Board and Jefferson County Department of Health. All women provided written informed consent.

## Supplementary Material

vlaf004_Supplementary_Data
