# Supplementary material for: Chlamydia trachomatis-specific interferon-γ-producing CD8 T-cells are associated with lower chlamydia bacterial load in reinfected women
Source: Immunohorizons. 2025 Apr 1;9(5):vlaf004. doi: 10.1093/immhor/vlaf004 (PMC11959114; doi:10.1093/immhor/vlaf004)
Supplement: vlaf004_Supplementary_Data [file vlaf004_supplementary_data.pdf]

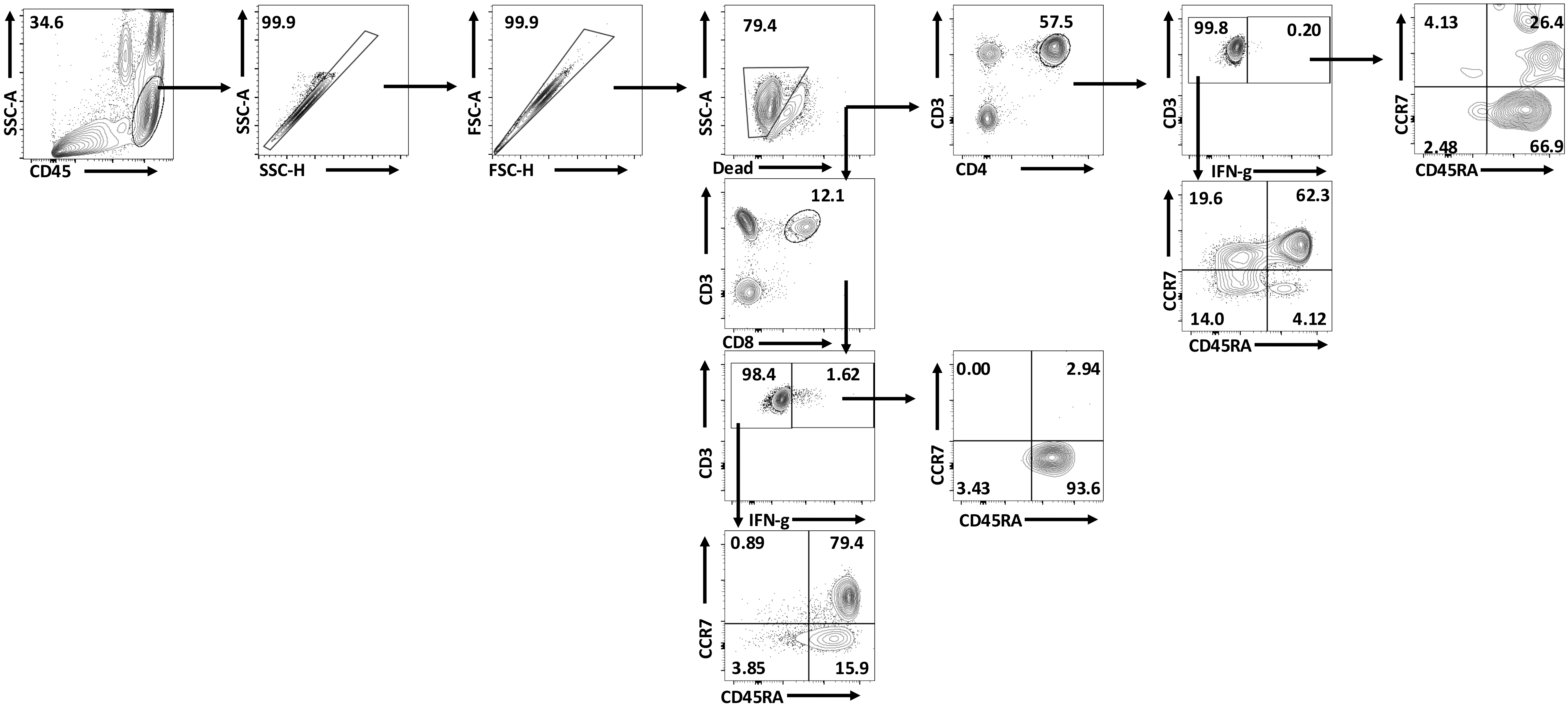

Supplementary Figure 1. Gating Strategy. Sample gating strategy for the percent CD3+CD4+ and CD3+CD8+ Memory T cell subsets and IFN-γ production for *Chlamydia trachomatis* MOMP 2 peptide pool from peripheral blood from a women seen at the 3-month follow-up visit after treatment for chlamydia . The percent gated population is shown above the gate.

Supplemental Table 1. Flow cytometry panels

Panel	Fluorophore	Conjugate	Clone	Manufacturer
Surface Markers	APC-eFluor 780	CD3	SK7	eBioscience
	BV786	CD4	SK3	BD Biosciences
	V500	CD8	RPA-T8	BD Biosciences
	Pe-CF594	CD45	HI30	BD Biosciences
	BV650	CCR7	2-L1-A	BD Biosciences
	BUV563	CD45RA	HI100	BD Biosciences
ICS	Pe-Cy7	TNFα	MAB11	BD Biosciences
	FITC	Granzyme B	GB11	BD Biosciences
	Alexa Fluor 700	IFN-γ	B27	BD Biosciences
